# A systems biology approach to the identification and analysis of transcriptional regulatory networks in osteocytes

**DOI:** 10.1186/1471-2105-10-S9-S5

**Published:** 2009-09-17

**Authors:** Angela K Dean, Stephen E Harris, Ivo Kalajzic, Jianhua Ruan

**Affiliations:** 1 Department of Computer Science, The University of Texas at San Antonio, San Antonio, TX 78249, USA; 2 Department of Periodontics, The University of Texas Health Science Center at San Antonio, San Antonio, TX 78229, USA; 3 Department of Cellular and Structural Biology, The University of Texas Health Science Center at San Antonio, San Antonio, TX 78229, USA; 4 Department of Reconstructive Sciences, University of Connecticut Health Center, Farmington, CT06030, USA

## Abstract

**Background:**

The osteocyte is a type of cell that appears to be one of the key endocrine regulators of bone metabolism and a key responder to initiate bone formation and remodeling. Identifying the regulatory networks in osteocytes may lead to new therapies for osteoporosis and loss of bone.

**Results:**

Using microarray, we identified 269 genes over-expressed in osteocyte, many of which have known functions in bone and muscle differentiation and contractility. We determined the evolutionarily conserved and enriched TF binding sites in the 5 kb promoter regions of these genes. Using this data, a transcriptional regulatory network was constructed and subsequently partitioned to identify *cis*-regulatory modules.

**Conclusion:**

Our results show that many osteocyte-specific genes, including two well-known osteocyte markers DMP1 and Sost, have highly conserved clustering of muscle-related *cis*-regulatory modules, thus supporting the concept that a muscle-related gene network is important in osteocyte biology and may play a role in contractility and dynamic movements of the osteocyte.

## Background

It is well known that bone tissue has the capacity to alter its mass and structure in response to mechanical strain. Osteocytes are terminally differentiated cells derived from osteoblasts, which first become embedded and surrounded by osteoid matrix that subsequently mineralizes [1]. They are regarded as the mechanosensory cells that respond to mechanical loading and a variety of hormones such as vitamin D and PTH, and sends signals to other bone cells to initiate bone formation and remodeling [1]. A better understanding of the gene networks regulating osteocytes can therefore lead to new therapies for osteoporosis, loss of bone in space travel and extended bed rest. However, even though osteocytes are the most abundant cells in bone, the regulatory pathways controlling osteocyte biology have not been identified.

As osteocytes are embedded within the bone matrix, with a complex network between the different stages of cells within the osteoblast-osteocyte lineage, studies of osteocytes have been hampered by their inaccessibility and by the lack of molecular and cell surface markers that could be used to isolate and characterize this cell population [2]. Dentin matrix protein (DMP-1) has been shown as a good marker for the osteocyte lineage and is specifically expressed along and in the canaliculi of osteocytes within the bone matrix, suggesting a role for DMP1 in osteocyte function. Recently, we generated a mouse model containing a DMP1 region, -7892 to +4439 bp (8 kb), driving GFP and thus directing expression to osteocytes [2]. This enables us to purify osteocytes from osteoblast cells using fluorescence-activated cell sorting, and compare the gene expression profiles in these two types of cells directly using microarray.

In this work, we developed a systems biology approach to study osteocyte biology by integrating data from microarray experiments, functional annotations and comparative genomics. This type of approaches has been shown to greatly eliminate noises contained in individual data sources, and improve the understanding of complex biological phenomena, such as Alzheimer's disease and cancer [3,4]. Typically, this type of approaches starts with identifying a set of differentially expressed genes, and then clusters genes according to their expression profiles or functions, followed by an analysis of *cis*-regulatory elements presented in the promoter sequences. Our method differs from those approaches in two important aspects. First, we only considered cis-regulatory elements that are both over-represented and evolutionary conserved. This significantly reduced the effective lengths of promoter regions when searching for *cis*-regulatory elements, and therefore eliminated many spurious matches. Moreover, we developed a graph theoretical method to identify transcriptional regulatory modules (CRMs) [5,6], which revealed interesting combinatorial relationships between several transcription factors.

Briefly, from microarray experiments, we obtained 269 osteocyte-specific genes, many of which have functions in bone or muscle development and contractility. We then identified enriched and evolutionarily conserved *cis*-regulatory elements from the 5 kb upstream promoter regions of a subset of 98 bone- and muscle-related genes, and used these data to construct a transcriptional regulatory network that links TFs to their putative binding sites on these 98 genes. We further proposed a graph-partitioning algorithm to identify possible *cis*-regulatory modules [5,6]. Our results show that many osteocyte-specific genes, including two well-known osteocyte markers DMP1 and Sost, have highly conserved clustering of muscle-related *cis*-regulatory modules, thus supporting the concept that a muscle-related gene network is important in osteocyte biology and may play a role in contractility and dynamic movements of the osteocyte.

## Results and discussion

### Bone and muscle-related genes are over-expressed in osteocyte cells

To identify potential regulatory networks of osteocytes, we obtained gene expression profiles from osteocytes purified from calvariae of 5–8 day-old mice expressing 8 kb DMP1 promoter driving GFP. As a control, we also obtained gene expression profiles from GFP-negative cells, which contain about 60% osteoblasts at different stages (before DMP1 gene turns on) and some macrophages. The microarray data is then normalized using GCRMA [7] and significantly differentially expressed genes were identified. We identified 269 genes that are over-expressed by at least 3 fold in osteocytes with a FDR-corrected p-value < 0.05 (See Methods).

Using the DAVID functional annotation tool [8], we found that the 269 osteocyte-specific genes are significantly enriched in several GO terms, which are further grouped into several functional clusters (Table 1). As expected, the most significant clusters include GO terms such as "extracellular region", "ossification", "bone remodelling" and "system development". Interestingly, our results also showed that osteocytes express many genes and transcription factors (TFs) known to control muscle differentiation and contractility. For example, over 12 myosin-related genes are over-expressed in osteocytes, as well as several TFs such as myogenin, Mef2c, and Myf5.

**Table 1:** Functional annotation clusters

**Annotation cluster**	**Enrichment score**	**SP_PIR_KEYWORD or GOTERM**	**# of genes**	**P-value**	**Benjamini corrected p-value**
1	12.69	GOTERM_CC: Extracellular region	83	1.6E-18	1.3E-15
		
		SP_PIR_KEYWORD: signal	88	4.3E-18	3.7E-15

2	11.72	GOTERM_BP: ossification	21	5.0E-19	2.6E-15
		
		GOTERM_BP: biomineral formation	21	6.3E-19	1.6E-15

3	10.37	GOTERM_BP: system development	65	5.8E-15	5.0E-12
		
		GOTERM_BP: anatomical structure development	71 71	7.4E-15	5.4E-12

4	6.34	GOTERM_CC: proteinaceous extracellular matrix	22	9.3E-10	1.5E-07
		
		GOTERM_CC: extracellular matrix	22	1.5E-09	2.0E-07

5	5.18	SP_PIR_KEYWORD: muscle protein	12	1.1E-11	3.1E-09
		
		GOTERM_BP: muscle system process	9	2.8E-05	5.0E-03

269 over-expressed in osteocyte cells were input into the DAVID Bioinformatics tool for functional annotation clustering. Sample clusters and their top two Protein Information Resource (SP_PIR) and GO terms are provided here, with Cluster 2 relating to bone and Cluster 5 relating to muscle.

### Conserved *cis*-regulatory elements in osteocyte-specific genes

To study the transcription regulation of the osteocyte-specific genes, we combined the 98 genes in the bone- and muscle-related functional clusters and analyzed the *cis*-regulatory elements occurring on their promoters. Using the web tool Whole Genome Vista (WGV) [9], we searched for known TF binding motifs that are conserved between the mouse and human genomes from the 5 kb promoter sequence upstream to the transcription starting site for each gene. As expected, many motifs identified are known to be related to bone and muscle functions, including myf2, Ets family, Smad3 sites, and FoxO1/4 *cis*-elements. Strikingly, 67 of the 98 genes had 10 or more conserved Mef2 binding sites in their 5 kb promoter regions. Furthermore, Mef2c is also a direct target of Mef2 with over 68 conserved binding sites, suggesting that this gene regulates itself (Table 2).

**Table 2:** Genes with Mef2 sites

Pthr1	20	Myl1	20	Myh8	22	Dlx3	21
Mef2a	37	Mylk	20	Myo1d	15	Phex	27
Myf5	31	Myh9	32	Myh11	13	Mepe	21
Myoz2	24	Myo1b	24	Tnnc2	13	Myf2c	68

Example of osteocyte and muscle related genes with high levels of conserved Mef2 binding sites. The number after each gene Symbol is the number of conserved Mef2 sites.

Modular structure of the transcriptional regulatory network

**Figure 1:**
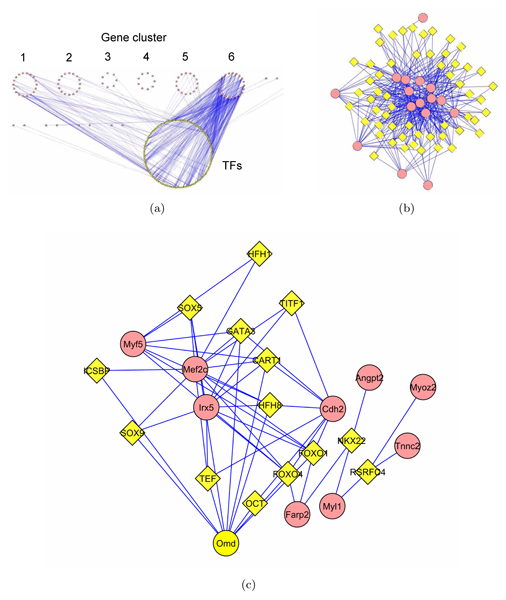
**Modular structures within the transcriptional regulatory network**. Yellow Triangles are the TFs and Red Circles are the target genes. (a) Clusters in the 98-gene and 153-TF network. (b) Cluster 6 contains 16 genes with a large set of common TF binding sites. (c) Cluster 1 showing potential co-regulation of Mef2c, Myf5, Irx5 by a common set of TF.

In order to identify possible combinatorial affects of TF binding sites, we created a transcriptional regulatory network including the 98 over-expressed genes and the known TF binding sites that are not only conserved between mouse and human, but are also determined by WGV to be enriched in some genes (i.e., their number of appearance on at least one of the 98 genes is statistically more significant than the rest of the genome). This increases the reliability of edges, while reducing the network size to a management size. A total of 153 over-represented TF motifs were identified for the 98 gene set. We created a network of these genes and TFs, with edges between genes and TFs representing an over-representation of that TF's binding site on that gene. We next applied Qcut [10], a spectral-based graph clustering algorithm for finding relatively dense subnetworks (aka communities in social sciences), to this initial regulatory network (see Methods). By optimizing a statistical score called the modularity, the Qcut function can determine by itself the most appropriate number of communities in the network. We identified 6 communities, each containing some genes that share a large set of common TF binding sites (Fig. 1(a)). Cluster 6 shows a strong community between 16 genes and their common TF binding sites, which is representative of many TFs coordinately regulating a small set of genes (Fig. 1(b)). Cluster 1 shows potential co-regulation of Mef2c, Myf5 and Irx5 by a common set of TFs (Fig. 1(c)).

### A putative model of the transcriptional network

**Figure 2:**
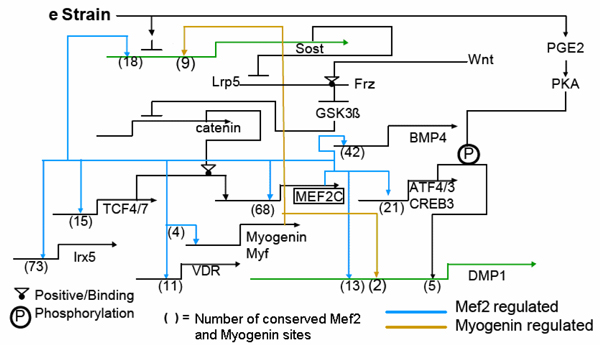
**Model of the potential role of Mef2c, Myogenin, and Creb family of TF in regulation of Sost and DMP1**. Prostaglandins and Pth can activate the Creb family of TF and regulate DMP1. DMP1 also has several Mef2 site and three are close to 2 myogenin sites. Myogenin and Mef2 synergize in activating several muscle related genes and possibly DMP1. Sost gene 5 kb region may also be regulated by Mef2 TF and Myogenin in this 5 kb region.

A proposed regulatory network model (Fig. 2) has been created using our network results and our prior biological knowledge. The model demonstrates the regulation of DMP1 and Sost, two genes highly expressed in osteocytes, by Mef2c and Myogenin. It is also observed that Mef2c contains a high level of Mef2c binding sites, suggesting that this gene regulates itself. These putative models can be used to generate hypotheses for experimental validation. We now have an ex vivo culture system for pure osteocytes in their proper microenvironment in which they make appropriate levels of osteocyte specific genes, and experiments from this model are currently underway.

## Conclusion

In this paper, we introduced a systems biology method for identifying and analyzing transcriptional regulatory networks in the osteocyte. We integrated data from microarray experiments, functional annotations, comparative genomics, and graph-theoretic analysis to create a putative model of the transcriptional regulatory networks in osteocytes. Many parts of the network can be confirmed by the literature, and more direct experimental validations are underway. Our model shows that many osteocyte-specific genes, including two well-known osteocyte markers DMP1 and Sost, have highly conserved clustering of muscle-related *cis*-regulatory modules, thus supporting the concept that a muscle-related gene network is important in osteocyte biology and may play a role in contractility and dynamic movements of the osteocyte.

## Methods

### Microarray experimental procedures and analysis

Three independent experiments were carried out with mice containing the DMP1-GFP transgene that marks osteocytes by the GFP expression. Following cell separation utilizing fluorescence activated cell sorting, the RNA was isolated from the GFP-positive and GFP negative cells. All experiments showed enrichment of 15 to 50 fold in DMP1 mRNA expression, a measure of osteocyte enrichment. The experiments with the 50 fold enrichment of osteocytes (GFP-positive) were focused on for this study with 3 replicate determinations of expression levels of all genes.

Microarray experiments were conducted using the Affymetrix 430A mouse chip with over 21,000 probes set. These raw .cel files are then normalized by GCRMA using Limma included in the Bioconductor package in R [7]. In these experiments we used the B statistic with B values greater than 3 and FDR = .05. We identified the top 269 genes out of the 21,000 that were differentially expressed between GFP-positive and GFP-negative cells.

### Deriving the 98 gene set related to bone/muscle

The top 269 differentially expressed genes were functionally clustered using the DAVID Bioinformatics tool, which also provides enrichment scores for each cluster [8]. For each GO term associated with a group of genes, a p-value is computed by the hypergeometric distribution, and then adjusted for multiple testing using the Benjamini method [8]. The enrichment score for a cluster is then calculated as the negative logarithm of the geometric mean of the individual GO p-values [8]. 98 of these 269 genes are functionally enriched in the skeletal/bone and muscle biology clusters with enrichment scores of 11.72 and 5.18, respectively.

### Building the transcriptional regulatory network

The 98 gene set was input into Whole Genome Vista [9] for discovery of conserved and over-represented TF binding sites occurring on the 5 kb upstream promoter sequence upstream to the transcription starting site of a gene. The motifs found by Vista are known motifs from the TRANSFAC database [11]. The significance of a motif found on a gene is determined by a p-value based on the number of occurrences of the motif in the 5 kb upstream promoter region of this gene as compared to the total number of occurrences of the motif in the same 5 kb region of the rest of the RefSeq genes in the genome. A potential regulatory network was created from this data in which an edge between a gene and a TF represents an over-representation of that TF's binding site on the gene's promoter, as according to WGV.

### Detecting network modules

In order to identify modules from the transcriptional regulatory network, we first assigned a cosine similarity score to each pair of genes according to their shared TFs. A weighted gene-gene network was then created in which an edge weight between two genes corresponds to their similarity score. This similarity matrix was then converted to a sparse network by connecting each gene to its k nearest neighbours (k = 7) with a similarity cutoff score equals 0.5. The network is then partitioned using the algorithm Qcut [10], resulting in gene sets that have many common TF binding sites. The regulatory network was input into Cytoscape [12] for visualization, along with the gene set partition information.

## Competing interests

The authors declare that they have no competing interests.

## Authors' contributions

AD implemented the program to collect data from rVista and performed the clustering, participated in the study's design and coordination, and participated in drafting the manuscript. SH participated in the study's design, coordination and conception, and carried out the osteocyte cell purification. IK prepared the biological samples for microarray experiments. JR participated in the study's design and coordination, and participated in drafting the manuscript. All authors read and approved the final manuscript.

## References

[B1] Yang W, Kalajzic I, Lu Y, Guo D, Harris MA, Gluhak-Heinrich J, Bonewald LF, Feng JQ, Rowe DW, Harris SE (2004). In vitro and in vivo study on osteocyte-specific mechanical signaling pathways. J Musculoskelet Neuronal Interact.

[B2] Kalajzic I, Braut A, Guo D, Jiang X, Kronenberg MS, Mina M, Harris MA, Harris SE, Rowe DW (2004). Dentin matrix protein 1 expression during osteoblastic differentiation, generation of an osteocyte GFP-transgene. Bone.

[B3] Yan B, Yang X, Lee T, Friedman J, Tang J, Van Waes C, Chen Z (2007). Genome-wide identification of novel expression signatures reveal distinct patterns and prevalence of binding motifs for p53, nuclear factor-κB and other signal transcription factors in head and neck squamous cell carcinoma. Genome Biol.

[B4] Ray M, Ruan J, Zhang W (2008). Variations in the transcriptome of Alzheimer's disease reveal molecular networks involved in cardiovascular diseases. Genome Biol.

[B5] Sharan R, Ovcharenko I, Ben-Hur A, Karp R (2003). CREME: a framework for identifying *cis*-regulatory modules in human-mouse conserved segments. Bioinformatics.

[B6] Ivan A, Halfon M, Sinha S (2008). Computational discovery of *cis*-regulatory modules in Drosophila without prior knowledge of motifs. Genome Biol.

[B7] Smyth GK (2004). Linear models and empirical Bayes methods for assessing differential expression in microarray experiments. Statistical Applications in Genetics and Molecular Biology.

[B8] Huang DW, Sherman BT, Tan Q, Collins JR, Alvord WG, Roayaei J, Stephens R, Baseler MW, Lane HC, Lempicki RA (2007). The DAVID gene functional classification tool: a novel biological module-centric algorithm to functionally analyze large gene lists. Genome Biol.

[B9] Loots GG, Ovcharenko I, Pachter L, Dubchak I, Rubin EM (2002). rVista for comparative sequence-based discovery of functional transcription factor binding sites. Genome Res.

[B10] Ruan J, Zhang W (2008). Identifying network communities with a high resolution. Physical Review E.

[B11] Matys V, Fricke E, Geffers R, Gössling E, Haubrock M, Hehl R, Hornischer K, Karas D, Kel AE, Kel-Margoulis OV, Kloos DU, Land S, Lewicki-Potapov B, Michael H, Münch R, Reuter I, Rotert S, Saxel H, Scheer M, Thiele S, Wingender E (2003). TRANSFAC: transcriptional regulation, from patterns to profiles. Nucleic Acids Res.

[B12] Shannon P, Markiel A, Ozier O, Baliga NS, Wang JT, Ramage D, Amin N, Schwikowski B, Ideker T (2003). Cystoscape: a software environment for integrated models of biomolecular interaction networks. Genome Res.

